# Fenmezoditiaz Inhibited the Acquisition and Transmission of Southern Rice Black-Streaked Dwarf Virus by *Sogatella furcifera*

**DOI:** 10.3390/insects16090875

**Published:** 2025-08-23

**Authors:** Yuting Chen, Lixin Mao, Xiulan Ding, Hengchien Liu, Devendra J. Vyas, Dongsheng Jia

**Affiliations:** 1 State Key Laboratory of Agriculture and Forestry Biosecurity, College of Plant Protection, Fujian Agriculture and Forestry University, Fuzhou 350002, China; cyt13194401680@outlook.com; 2 BASF Corporation, 26 Davis Drive, Research Triangle Park, Durham, NC 27709, USA; lixin.mao@basf.com; 3 BASF (China) Company Ltd., 300 Jiang Xin Sha Road, Pu Dong, Shanghai 200137, China; linda.ding@basf.com; 4 BASF South East Asia Pte Ltd., Singapore 189773, Singapore; derrick.liu@basf.com (H.L.); devendra.vyas@basf.com (D.J.V.)

**Keywords:** fenmezoditiaz, *Sogatella furcifera*, electrical penetration graph, feeding behaviors, viral transmission

## Abstract

Rice planthoppers are the most destructive pests of rice production and the vectors of rice viruses. Fenmezoditiaz as a novel mesoionic insecticide is used for rice planthopper management by targeting the insect’s neural nicotinic acetylcholine receptor. This study aimed to evaluate the effects of fenmezoditiaz on the acquisition, propagation, and transmission of southern rice black-streaked dwarf virus (SRBSDV) by the white-backed planthopper, *Sogatella furcifera* (Hemiptera: Delphacida). The results revealed that sublethal concentrations of fenmezoditiaz significantly impaired SRBSDV acquisition and viral replication in *S. furcifera*. Fenmezoditiaz-treated viruliferous *S. furcifera* exhibited a lower transmission efficiency of SRBSDV to un-infected rice seedlings. Electrical penetration graph (EPG) recordings revealed prolonged non-probing (NP), salivary secretion (N2/N3), and xylem feeding (N5) durations, alongside shortened phloem contact behavior (N4a/N4b), of fenmezoditiaz-treated individuals, indicating disrupted feeding behaviors, which are critical for reducing viral infection. By reducing viral titers and interfering with phloem ingestion, fenmezoditiaz significantly suppresses SRBSDV transmission. These findings revealed fenmezoditiaz’s dual role in pest control and viral transmission blockage, providing a foundation for incorporation into integrated management of vector-borne plant viruses.

## Simple Summary

White-backed planthoppers are serious rice pests that are also the vector of a harmful southern rice black-streaked dwarf virus. Fenmezoditiaz, as a novel mesoionic insecticide, is used for rice planthopper management by targeting the insect’s neural nicotinic acetylcholine receptor. In this study, the sublethal concentrations of fenmezoditiaz inhibited the acquisition and transmission of southern rice black-streaked dwarf virus by influencing feeding behaviors of white-backed planthopper. These revealed fenmezoditiaz’s dual role in pest control and viral transmission blockage, providing a foundation for incorporation into integrated management of vector-borne plant viruses.

## 1. Introduction

Rice is one of the most important food crops in the world. However, stable rice production is constrained by various diseases and pests [1]. With the increasing pressure on global food security, the effective detection and management of rice pests and diseases have become crucial [2]. Rice planthoppers (including *Nilaparvata lugens*, *Sogatella furcifera*, and *Laodelphax striatellus*) are the most destructive pests of rice production in Asia. Rice planthoppers not only suck rice sap and oviposit in rice tissues, but they also act as vectors of five major rice viruses and two wheat viruses [3,4]. Southern rice black-streaked dwarf virus (SRBSDV), transmitted by the white-backed planthopper *S. furcifera* in a persistent propagative manner, is one of the most destructive viruses on rice and has inflicted severe yield losses and compromised the quality of rice across Asia over the last 20 years [5,6,7]. As the area affected by the disease is over 1.3 million hectares, the yield loss reaches over 30%, and it is necessary for the Ministry of Agriculture and Rural Affairs to coordinate, guide, and organize control; southern rice black-streaked dwarf disease was classified as one of eight category-I diseases defined by the Ministry of Agriculture and Rural Affairs of China.

Although various strategies have been developed to control planthoppers and prevent the occurrence of viral diseases, application of chemical insecticides is still the main method used [8,9]. However, the misuse and overuse of chemical insecticides against rice planthoppers have resulted in the development of significant levels of resistance to insecticides, such as imidacloprid, thiamethoxam, dinotefuran, chlorpyrifos, pymetrozine, and buprofezin [10,11,12]. Thus, the development of new insecticides that are highly effective against resistant rice hopper populations and more environmentally friendly is urgently needed. Recently, a novel mesoionic insecticide, fenmezoditiaz, was reported, targeting the insect neural nicotinic acetylcholine receptor (nAChR) with a broad spectrum of activity, especially for the rice planthopper complex [13]. It has been added to the Insecticide Resistance Action Committee mode of action classification for plant protection against piercing–sucking pests.

Insecticides not only kill insects directly with lethal doses, but they can also elicit behavioral changes in the survivors exposed to sublethal doses [14]. Sublethal concentrations of insecticides in natural agro-ecosystems are common as insecticides degrade post-application. These sublethal levels, while not causing significant pest mortality, can induce physiological and/or behavioral changes in insects [15]. The behavior of insects is largely governed by neural interactions [16], and most synthetic insecticides target the insect nervous system through various mechanisms, interfering with neural transmission and causing abnormal behaviors of dispersal, locomotion, reproduction, feeding, and host-finding in insects [17,18,19,20]. In particular, the effect of insecticides on feeding behavior of insects can directly destroy their ability to transmit plant viruses. For example, flonicamid limited the ability of *Myzus persicae* to acquire turnip yellows virus by reducing phloem contact and sap ingestion [21]. Flupyradifurone and dinotefuran limited the ability of *Bemisia tabaci* to transmit tomato chlorosis virus through disrupting phloem activity [22]. The effect of the new insecticide fenmezoditiaz on the ability of *S. furcifera* to transmit SRBSDV is still unknown.

To understand the impact of fenmezoditiaz on *S. furcifera*’s ability to transmit SRBSDV, in this study, we first determined the sublethal concentrations of fenmezoditiaz to *S. furcifera* by rice-seedling dip and topical application methods, which allowed for measuring the effects of SRBSDV acquisition, propagation, and transmission by *S. furcifera*. Feeding behavior is an important factor influencing the acquisition and transmission of plant viruses by vector insects. Hence, the feeding behaviors of *S. furcifera* treated with fenmezoditiaz were investigated to account for the effects of fenmezoditiaz on the transmission of SRBSDV. The results would help to further clarify the importance of fenmezoditiaz on management of *S. furcifera* and blocking the spread of SRBSDV disease.

## 2. Materials and Methods

### 2.1. Insect, Virus, and Antibodies

Nonviruliferous individuals of *S. furcifera* were collected from Shunchang, Fujian, China (26°47′35″ N 117°48′36″ E), and subsequently propagated in insect cages (50 cm length × 40 cm width × 40 cm height) at 25 ± 3 °C on un-infected rice seedlings (~15 cm tall) with 75 ± 5% relative humidity and a 16 h light/8 h dark–light cycle [23]. Rice plants infected with SRBSDV were also collected from fields in Shunchang, Fujian, China. Antibodies against P10 of SRBSDV were used to detect the accumulation level of SRBSDV [23]. The rice variety Taichung Native 1 (TN-1) was used for all the tests. The fenmezoditiaz suspension concentrate formulation was provided by BASF (China) Company Ltd. (Shanghai, China).

### 2.2. Determination of Sublethal Concentrations of Fenmezoditiaz

Second-instar nymphs of *S. furcifera* were treated with a rice-seedling dip method that has been described previously [24]. Briefly, fifteen rice seedlings (~15 cm tall) as a group were immersed in 0, 0.2, 0.3, or 0.4 mg/L fenmezoditiaz containing 0.1% Dimethyl sulfoxide (DMSO) for 60 s and then air-dried at 25 °C for 30 min. The roots of rice seedlings were placed in a glass cup filled with water in insect cages (20 cm length × 20 cm width × 20 cm height). Next 100 second-instar nymphs were introduced onto the treated seedlings. Three replicates of each concentration were carried out. Mortality was counted after exposure to fenmezoditiaz for 2, 4, 6, 12, 24, 36, and 48 h. Additionally, un-infected *S. furcifera* (fifth-instar nymphs or adults) were treated by topical application on the pronotum with different doses (applying 0.069 µL droplet/individual of 0, 0.2, 0.3, or 0.4 mg/L solutions by a micro syringe) of fenmezoditiaz and then placed on un-infected rice seedlings (~15 cm tall) to observe insect activity and mortality over time. In topical application, DMSO facilitates rapid penetration of pesticides through the insect cuticle, ensuring the chemicals reach target sites. In the rice-seedling dip method, it enhances pesticide absorption via leaf surfaces. Compared with 0 mg/L fenmezoditiaz-treated *S. furcifera*, the concentration of fenmezoditiaz that can cause approximately 20% mortality of *S. furcifera* was calculated.

### 2.3. Influence of Fenmezoditiaz on SRBSDV Acquisition Ability of S. furcifera

Second-instar *S. furcifera* (the most effective instar for acquiring virus) were fed on 0 or 0.2 mg/L fenmezoditiaz-treated rice seedlings (dip method) for 24 h, then transferred to feed on SRBSDV-infected rice for 1 day. Sequentially, the insects were placed on un-infected rice seedlings (~15 cm tall) for 6 days. Next, total RNA was extracted from individual insects using TRIzol reagent (Invitrogen, Waltham, MA, USA) according to the manufacturer’s protocol. The individual extracts of 100 *S. furcifera* were used to detect the SRBSDV P10 gene in a reverse transcription–polymerase chain reaction (RT-PCR) assay. The percentage of the RT-PCR-positive result was used to calculate the acquisition rates of SRBSDV by *S. furcifera*. The experiments were repeated ten times independently.

### 2.4. Influence of Fenmezoditiaz on the Propagation of SRBSDV in S. furcifera

To observe the effect of fenmezoditiaz on viral propagation in insects, 100 second-instar *S. furcifera* were fed on insecticide-treated or not-treated rice seedlings (dip method) for 24 h and then fed on SRBSDV-infected rice plants for 1 day. Sequentially, the insects were placed on un-infected rice seedlings. The SRBSDV genome copy numbers in the individual midguts from insecticide-treated or not-treated viruliferous *S. furcifera* were detected by an absolute RT-qPCR assay, as described previously [25]. Briefly, total RNA was extracted from individual insects and reverse transcribed using RevertAid reverse transcriptase (Promega, USA, Madison, WI, USA), following the manufacturer’s instructions. An SYBR Green PCR Master Mix kit (Promega, Madison, WI, USA) was used to detect the transcript levels of SRBSDV P10 in viruliferous insects by an RT-qPCR assay. The number of copies per microgram of midgut RNA were calculated by mapping the CT value to the standard curve for the P10 gene of SRBSDV [25]. At 6 days post-first access to diseased plants (padp), the expression of the SRBSDV P10 gene in 50 *S. furcifera* was detected by RT-PCR assay. An SYBR Green PCR Master Mix kit (Promega, Madison, WI, USA) was used to detect the transcript levels of SRBSDV P10 in viruliferous insects by an RT-qPCR assay. The actin transcript of *S. furcifera* served as the internal reference and the relative gene expression levels were calculated using the 2^−ΔΔCT^ method [26]. Meanwhile, the total proteins from 50 intact insects fed on insecticide-treated or not-treated rice seedlings were extracted and the accumulation levels of the SRBSDV P10 protein were detected by a Western blot assay with P10-specific IgGs. In addition, the internal organs of 30 *S. furcifera* treated with insecticide or not were dissected, fixed in 4% paraformaldehyde, permeabilized with 1% Triton X-100, and immunostained with P10-FITC and actin-rhodamine, as described previously [27], and then examined with a Leica TCS SP5 inverted confocal microscope (Leica, Wetzlar, Germany).

### 2.5. Influence of Fenmezoditiaz on Inoculation Rate of SRBSDV by S. furcifera

The 3rd instar nymphs of *S. furcifera* were allowed to feed on rice plants infected with SRBSDV for 2 days to acquire the virus and then transferred to un-infected rice plants. At 10 days padp, 100 insects were topically treated with 0.069 μL/insect of either 0 or 0.2 mg/L fenmezoditiaz solution. One day later, 50 live insects were transferred to 50 un-infected rice seedlings (2-leaf stage) individually. The rice seedlings were replaced daily with un-infected ones for 12 days. About 600 rice seedlings were used in each replication. This setup was repeated 10 times. The replaced rice seedlings were grown in the greenhouse for about 15 days to observe the appearance of disease symptoms, and then the total RNA was extracted from rice seedlings inoculated with viruliferous *S. furcifera* to determine the presence of the SRBSDV P10 gene to calculate inoculation rates as the number of disease-positive rice plants divided by the total number of rice plants fed by insecticide-treated hoppers. The transcript levels of SRBSDV P10 in the individual rice seedling after being fed by insecticide-treated or not-treated viruliferous *S. furcifera* for 15 days were detected by RT-qPCR as described above. All the experiments were repeated ten times independently.

### 2.6. Influence of Fenmezoditiaz on Feeding Behaviors of S. furcifera Recorded by Electrical Penetration Graphs

A Giga-8 DC-EPG device with 1 GΩ input resistance (EPG Systems, The Netherlands) was used to monitor the feeding activities of insecticide-treated or not-treated *S. furcifera* on rice, as described previously [28,29]. Briefly, 50 fifth-instar or adult viruliferous *S. furcifera* (after acquiring SRBSDV for 10 days) were topically applied with either a sublethal dose of 0.2 mg/L fenmezoditiaz (0.069 µL/insect) or 0 mg/L fenmezoditiaz solution as a control and fed on untreated rice seedlings for one day before EPG recordings. After starving for 1.5 h, one end of a gold wire was attached to the dorsal thorax of the *S. furcifera* using a drop of water-soluble silver glue and connected to the EPG input probe and placed on a rice leaf sheath of a potted plant. A copper wire, connected to the amplifier and vertically inserted into the pot soil, was used as the plant electrode. The EPG signals were digitized with a converter (DI710-UL, Dataq, Akron, OH, USA), and the gain of the amplifier was recorded and stored with the PROBE 3.4 software (Wageningen University, Wageningen, The Netherlands). Each insect was continuously recorded for 6 h. Both insects and plants were used only once and then discarded. Recordings were made simultaneously on six plants placed within a Faraday cage at 27 ± 2 °C under electric fluorescent lighting. For each waveform observed in insecticide-treated or not-treated *S. furcifera*, a total of 30 insects that exhibited the reported waveform were recorded. Immediately after each EPG recording, the virus infection status of the test insects was individually assessed by RT-PCR, and positive insects were considered as valid samples and included in the final analysis.

### 2.7. Statistical Analysis

The insect mortality rate was calculated using the following formula: P = K/N × 100%. K: number of dead insects; N: total number of insects tested. The insect mortality data are presented as “mean ± standard error of mean (SEM).” Statistical analysis was performed using SPSS 22.0 (SPSS Inc., Chicago, IL, USA) for one-way ANOVA for multiple comparisons to determine significant differences (*p* < 0.05). Statistical analyses of viral acquisition, titer, propagation, transmission, and total duration of different waveform data were performed using SPSS 22.0 (SPSS Inc., Chicago, IL, USA) for unpaired data or multiple *t* tests to determine significant differences. Graphs were generated using GraphPad Prism 8 software (GraphPad Software, San Diego, CA, USA).

## 3. Results

### 3.1. Sublethal Concentrations of Fenmezoditiaz

**Figure 1:**
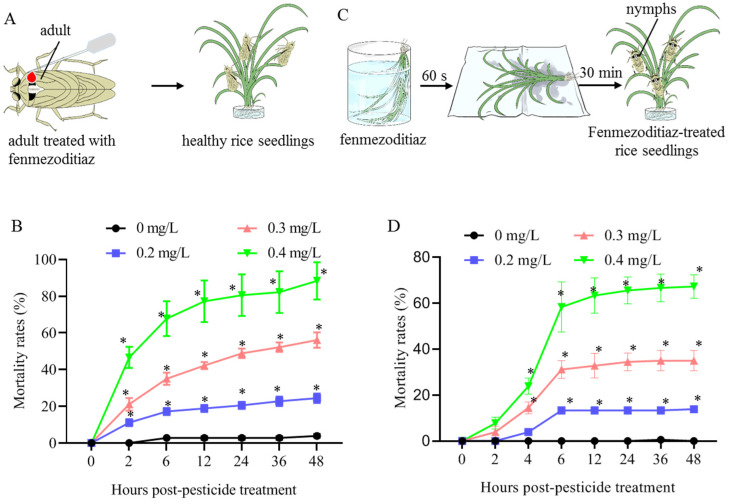
Determination of sublethal concentrations of fenmezoditiaz. (**A**,**B**) Effects of 0.069 μL/insect of 0, 0.2, 0.3, or 0.4 mg/L fenmezoditiaz on mortality of *S. furcifera* treated with topical application method. (**C**,**D**) Effects of 0, 0.2, 0.3, or 0.4 mg/L fenmezoditiaz on mortality of *S. furcifera* treated with rice-seedling dip method. Means (±SEM) in (**B**,**D**) were shown from three biological replicates (one-way ANOVA). *, *p* < 0.05.

In this study, for the purpose of measuring the effect of fenmezoditiaz on virus transmission, the sublethal concentrations of fenmezoditiaz that cause ~20% mortality was determined using the rice-seedling dip and topical application methods (Figure 1). The results showed that the mortality of *S. furcifera* second-instar nymphs treated with 0.069 μL/insect of 0, 0.2, 0.3, or 0.4 mg/L fenmezoditiaz topically reached 3.5 ± 0.004%, 24.5 ± 0.096%, 56.1 ± 0.013%, and 88.3 ± 0.087%, respectively, at 48 h after treatment (Figure 1A,B).The mortality of *S. furcifera* treated with 0, 0.2, 0.3, or 0.4 mg/L fenmezoditiaz by the rice-seedling dip method was 0, 13.9 ± 0.14%, 35.1 ± 0.012%, and 67.2 ± 0.013%, respectively, at 48 h post treatment (Figure 1C,D). Within 30 min of treatment, by lightly touching them with a brush, *S. furcifera* in the 0.3 and 0.4 mg/L fenmezoditiaz groups showed obvious poisoning symptoms and exhibited a higher mortality rate. Some *S. furcifera* in the 0.2 mg/L fenmezoditiaz group began to display slow crawling activity and a tendency to congregate near the rice stems. Two hours later, most *S. furcifera* had recovered basic mobility, but when lightly probed, their reaction ability was still relatively sluggish compared to that of the control group, and their feeding activity subsequently became concentrated on the leaf blade, rather than on the leaf sheath. These observations characterized the quick impact of fenmezoditiaz on *S. furcifera* behaviors. The mortality of *S. furcifera* treated with 0.2 mg/L fenmezoditiaz in both methods was less than 25% after 48 h, thus this concentration was chosen as the dose for subsequent virus transmission tests.

### 3.2. Fenmezoditiaz Reduced the Acquisition and Propagation of SRBSDV in S. furcifera

**Figure 2:**
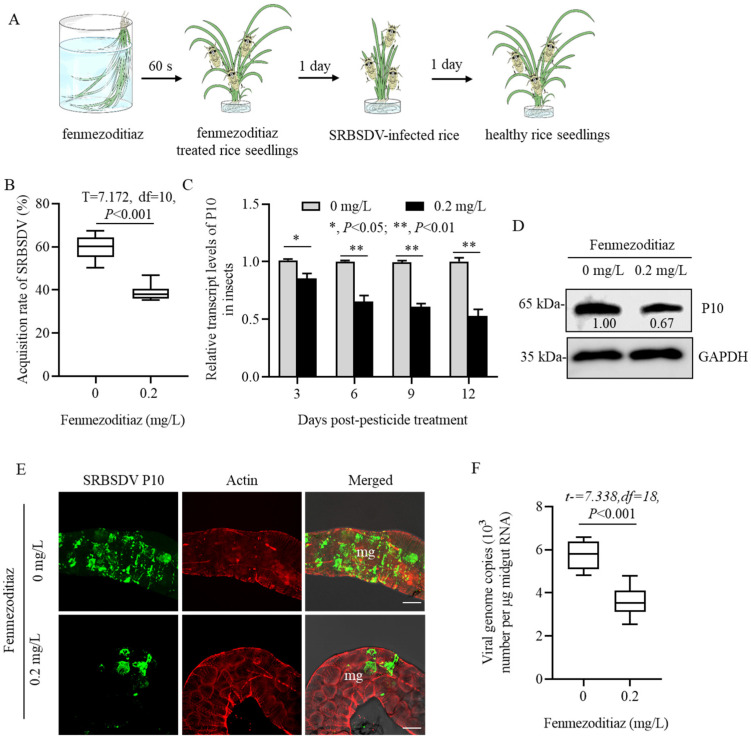
Fenmezoditiaz reduces the SRBSDV acquisition ability and inhibits virus propagation in *S. furcifera*. (**A**) An acquisition assay for investigating the effect of fenmezoditiaz-dipped rice seedlings on the SRBSDV acquisition ability of second-instar *S. furcifera*. (**B**) Fenmezoditiaz significantly reduced the viral acquisition efficiency of *S. furcifera*. (**C**) The relative transcript levels of SRBSDV P10 in insecticide-treated or not-treated *S. furcifera* detected by RT-qPCR assay. (**D**) The accumulation levels of SRBSDV P10 in insecticide-treated or not-treated *S. furcifera* detected by a Western blot assay. (**E**) Immunofluorescence assay showing the distribution of SRBSDV P10 in midgut epithelial cells of *S. furcifera* fed on 0 or 0.2 mg/L fenmezoditiaz-dipped rice seedlings. At 6 days padp, the intestines of *S. furcifera* individuals were immunostained with P10-FITC (green) and actin dye phalloidin-Alexa Fluor 647 (red) and then examined by immunofluorescence microscopy. mg, midgut. Bars, 5 μm. (**F**) Viral genome copies in individual midguts of insecticide-treated or not-treated viruliferous *S. furcifera* were detected with absolute RT-qPCR assays. Means (±SEM) in (**B**,**F**) were shown from ten biological replicates (unpaired *t* test, two-tailed). Means (±SEM) in C were shown from six biological replicates (multiple *t* test, two-tailed). *, *p* < 0.05; **, *p* < 0.01.

To determine the effect of fenmezoditiaz on the acquisition and propagation of SRBSDV, the second-instar *S. furcifera* were allowed to feed on insecticide-treated rice and then SRBSDV-infected rice for 1 day and were finally transferred to un-infected rice plants (Figure 2A). At 6 days post-first access to diseased plants (padp), the RT-PCR assay showed that the number of SRBSDV-positive *S. furcifera* in the fenmezoditiaz-treated group was significantly less than that in the control group (DMSO) (Figure 2B). The RT-qPCR analysis also revealed that the transcript levels of SRBSDV P10 in the fenmezoditiaz-treated *S. furcifera* were significantly less than those in the DMSO-treated *S. furcifera* at 3, 6, 9, and 12 days padp (Figure 2C). Western blot assays showed that fenmezoditiaz treatment decreased the accumulation levels of SRBSDV P10 (Figure 2D). Immunofluorescence labeling further confirmed that the fenmezoditiaz treatment significantly reduced the infection of SRBSDV in the midgut epithelial cells of the insects (Figure 2E). To further determine the reason for the low accumulation of SRBSDV P10 in fenmezoditiaz-treated *S. furcifera*, the virus load in the insects that were feeding on SRBSDV-infected rice for 1 day was detected by the absolute RT-qPCR assay. It was found that the virus content in insecticide-treated *S. furcifera* was significantly reduced to 65% of that in the control group (*p* < 0.001) (Figure 2F). These results suggest that fenmezoditiaz reduced the acquisition ability and titer of SRBSDV, thus inhibiting the propagation of SRBSDV in *S. furcifera*.

### 3.3. Fenmezoditiaz Reduced the Inoculation Rate of SRBSDV by S. furcifera

**Figure 3:**
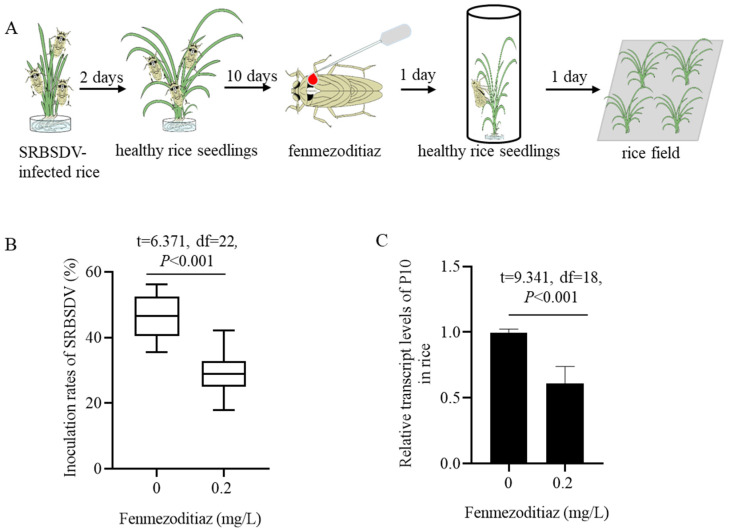
Transmission rates of SRBSDV by viruliferous *S. furcifera* treated with fenmezoditiaz. (**A**) Viral transmission scheme used to investigate the effect of fenmezoditiaz on the transmission ability of viruliferous adult *S. furcifera*. (**B**) Transmission rates of SRBSDV by viruliferous *S. furcifera* after being treated with 0 or 0.2 mg/L fenmezoditiaz. (**C**) The relative transcript levels of SRBSDV P10 in viruliferous rice seedlings transmitted by insecticide-treated or not-treated *S. furcifera* after 15 days, detected by an RT-qPCR assay. Means (±SEM) in (**B**,**C**) were shown from ten biological replicates (unpaired *t* test, two-tailed).

Viruliferous adult *S. furcifera* were topically treated with fenmezoditiaz and individually fed on un-infected rice seedlings for 12 days, with seedlings replaced daily and then transferred to the field (Figure 3A). After 15 days of growth in the field, an RT-PCR assay was used to detect the SRBSDV-positive rate of the rice seedlings. The results showed that the inoculation rates of SRBSDV in fenmezoditiaz-treated viruliferous adult *S. furcifera* were significantly lower than those in the insecticide not-treated control (Figure 3B). Meanwhile, RT-qPCR assay showed that the transcript levels of SRBSDV P10 in positive rice seedlings inoculated by fenmezoditiaz-treated *S. furcifera* were lower than those inoculated by not-treated *S. furcifera* (Figure 3C). These results suggest that fenmezoditiaz reduced the inoculation rate of SRBSDV by *S. furcifera*, thereby weakening the spread of SRBSDV.

### 3.4. Fenmezoditiaz Affects the Feeding Behavior of S. furcifera

**Figure 4:**
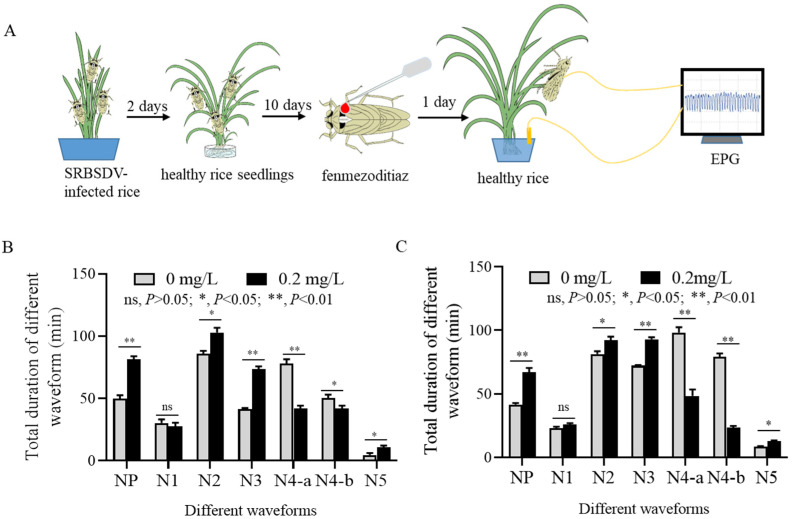
Effect of fenmezoditiaz on the feeding behaviors of *S. furcifera*. (**A**) EPG setup used to investigate the effect of fenmezoditiaz on feeding behaviors of viruliferous *S. furcifera* on rice plants. (**B**) EPG waveforms produced by insecticide-treated or not-treated fifth-instar nymphs of viruliferous *S. furcifera* feeding on rice plants. (**C**) EPG waveforms produced by insecticide-treated or not-treated viruliferous adult *S. furcifera* feeding on rice plants. NP, non-penetration of stylets; N1, penetration initiation; N2, salivation and stylet movement; N3, extracellular activity near the phloem region; N4a, intracellular phloem contact; N4b, phloem sap ingestion; N5, activity in the xylem region. Means (±SEM) in (**B**,**C**) were shown from three biological replicates with 10 insects recorded in each replication (multiple *t* test, two-tailed). *: *p* < 0.05; **: *p* < 0.01; ns: *p* > 0.05.

We used an electrical penetration graph (EPG) assay to investigate whether fenmezoditiaz affects the feeding behavior of viruliferous *S. furcifera* on rice plants (Figure 4A). The recordable EPG waveform patterns were similar between insecticide-treated or not-treated fifth-instar nymphs or adult viruliferous *S. furcifera*. There were seven EPG waveforms corresponding to specific feeding behaviors: the non-penetration phase (NP), initial penetration (N1), salivary secretion with stylet probing (N2), extracellular activity adjacent to phloem (N3), intracellular phloem contact (N4a), phloem sap ingestion (N4b), and stylets in the xylem tissue (N5). The total duration of the NP, N2, N3, and N5 waveforms was significantly prolonged by insecticide treatment compared to the control, while the N1 and N5 waveforms were not significantly different from the control. On the other hand, fenmezoditiaz significantly shortened the total duration of N4a and N4b (Figure 4B). The effect of fenmezoditiaz on the total duration of different waveforms of adult viruliferous *S. furcifera* was similar to that of fifth-instar nymph *S. furcifera* (Figure 4C). These results indicated that fenmezoditiaz treatment potentially caused viruliferous *S. furcifera* to rest for longer periods, require more probing attempts during feeding and encountering more obstacles while reaching the phloem. As a result, viruliferous *S. furcifera* reduced salivary secretion to the phloem, which agrees with the results that the inoculation rates of SRBSDV by insecticide-treated viruliferous *S. furcifera* are less than those of not-treated viruliferous *S. furcifera*.

## 4. Discussion

The present study demonstrates that fenmezoditiaz significantly impairs the acquisition, propagation, and transmission of SRBSDV by its insect vector, *S. furcifera*, while simultaneously disrupting the feeding behavior of *S. furcifera*. The impairment of viral transmission and disruption of feeding behavior may be inter-related. The transmission of phloem-infected plant viruses depends on sustained phloem contact. Since SRBSDV transmission primarily occurs during sustained phloem feeding, the behavioral modifications in *S. furcifera* are critical for SRBSDV transmission efficiency [7]. The suppression of SRBSDV transmission by fenmezoditiaz-treated *S. furcifera* likely stems from altered feeding behavior which limits viral release into the phloem. The prolonged non-penetration (NP) and probing phases (N2, N3), coupled with shortened phloem contact (N4a/N4b), indicate that fenmezoditiaz-treated vectors encounter barriers that delay salivation and reduce phloem ingestion (Figure 4B,C). Furthermore, fenmezoditiaz-induced neurotoxicity potentially affected stylet coordination and impaired vector mobility and feeding activity, which is similar to the effects of sublethal doses of insecticides pymetrozine, imidacloprid, and sulfoxaflor [28,30]. Similar behavioral disruptions, including prolonged probing and reduced phloem ingestion, have been documented in *Bemisia tabaci* exposed to cyantraniliprole [31]. The findings of this study also align with previous reports on the sublethal effects of insecticides, which can reduce vector competence by altering pathogen–vector interactions [15,32] and underscores the importance of evaluating sublethal insecticide effects to fully exploit their potential in combating vector-borne plant viruses. In addition to altered feeding behavior, the reduction of SRBSDV acquisition and replication in fenmezoditiaz-treated *S. furcifera* suggests that the insecticide interferes with early viral entry or replication mechanisms. This effect may be attributed to a reduced viral load in the midgut of fenmezoditiaz-treated *S. furcifera* (Figure 2F), lowering the likelihood of infection in the midgut during feeding. Previous reports showed that piperazine derivatives prevent the assembly of the virus core structure ribonucleoprotein (RNP) of tomato spotted wilt virus [33]. Whether fenmezoditiaz interferes with viral assembly or spread within *S. furcifera* to reduce the accumulation of SRBSDV is unknown. Future research should explore the modulation of insect immune genes or direct antiviral activity underlying fenmezoditiaz-induced viral suppression and validate these findings in field conditions.

As a newly discovered insecticide, fenmezoditiaz has a broad insecticidal spectrum for pest management [13]. The findings in this study reveal the dual utility of fenmezoditiaz in integrated pest management strategies, targeting both pest population suppression and disruption of viral transmission dynamics. Such a property positions fenmezoditiaz as a valuable tool for managing rice hoppers and SRBSDV outbreaks by reducing both vector populations and transmission efficiency, as well as delaying the epidemic development of both the vector and the virus. As virus diseases gain importance in rice production, fenmezoditiaz will play an important role in supporting rice growers.

## Data Availability

The original contributions presented in the study are included in the article, further inquiries can be directed to the corresponding author.

## Abbreviations

EPG	electrical penetration graph
DMSO	Dimethyl sulfoxide
SRBSDV	southern rice black-streaked dwarf virus
RT-PCR	reverse transcription–polymerase chain reaction
padp	post-first access to diseased plants
